# Advanced perfusion strategy for renal protection in juxtarenal aortic aneurysms: a pilot study

**DOI:** 10.1186/s12872-026-05549-7

**Published:** 2026-01-28

**Authors:** Melanie Rusch, Grischa Hoffmann, Nawar Alasad, Rene Rusch

**Affiliations:** 1 Clinic of Vascular and Endovascular Surgery, University Hospital of Schleswig-Holstein, Campus Kiel, Kiel, Germany; 2 University Vascular Center North, University Hospital of Schleswig-Holstein, Campus Kiel, Kiel, D-24105 Germany

**Keywords:** Renal ischemia, Organ protection, Open aortic repair, Heart-lung machine, Acute kidney injury, Juxtarenal aortic aneurysm

## Abstract

**Objectives:**

Open surgical treatment of juxtarenal aortic aneurysms (JAAA) is often associated with acute kidney injury (AKI). Therefore, intraoperative organ protection during supra-renal clamping is decisive for the outcome. This study describes the use of a low-profile extracorporeal circulation (LPECC) for selective renal perfusion in open surgery of JAAA.

**Methods:**

From 2018 to 2024, 23 patients with JAAA underwent open aortic repair with suprarenal cross-clamping with organ protection by LPECC. This retrospective case series without control group investigated the effect of pressure- and volume-controlled renal perfusion during clamping on clinical outcome in terms of prevention of AKI after open surgery. To classify postoperative renal dysfunction, the RIFLE classification (risk, injury, failure, loss, end-stage renal disease) was used. AKI was defined in the postoperative course as a decrease in eGFR of more than 50% (RIFLE class ≥ 2). Renal function was monitored after 30-days and 1-year with regard to the need for dialysis, course of renal parameters and the occurrence of complications.

**Results:**

The intraoperative use of the LPECC was not associated with any intraoperative complications. In the postoperative course, 17% developed temporary AKI (RIFLE class ≥ 2, *n* = 4) of which 3 patients required dialysis. During 30-days and 1-year follow-up, the retention values recovered and decreased to the preoperative level. None of the investigated patients required long-term dialysis.

**Conclusions:**

Monitored pressure- and volume-controlled renal perfusion could improve management and outcome in patients with JAAA. LPECC represents a safe and feasible surgical method for renal protection.

## Introduction

Open surgical treatment of aortic pathologies, in particular juxtarenal aortic aneurysms (JAAA), are a rare but life-threatening disease and account for approximately 15% of abdominal aortic aneurysms (AAA) [1–3]. Despite the successes of endovascular procedures, open repair continues to be an important treatment option, with regard to the current UK-COMPASS study. Nevertheless, open surgery in JAAA is technically challenging in terms of unavoidable reduced renal perfusion during cross-clamping and associated with an increased rate of postoperative complications [3–5]. Morbidity and mortality rates are reported in the literature between 5% and 30% mainly driven by AKI [4, 6–8]. The individual components that lead to AKI in the postoperative course are complex and have not yet been fully explored in well-designed studies [9]. Several studies demonstrated that 30-day mortality is significantly increased in cases of postoperative AKI [10, 11]. The use of crystalloid solution (containing mannitol) or cold blood are general accepted methods for temporary selective renal perfusion and protection [12, 13]. The European Society of Vascular Surgery (ESVS Guidelines) currently recommend alternative renal perfusion for open repair of complex aortic aneurysms with suprarenal clamping times of > 25 min [14]. However, the efficiency of selective perfusion continues to be controversially discussed [15, 16].

A high percentage of patients with suprarenal aortic pathologies have prescribed renal disease which further increases the risk of AKI [6, 17]. Suprarenal cross-clamping could lead to renal tubular injury, which can be aggravated by postoperative volume shift and postoperative complications e.g. bleeding complications and cardiac failure [13, 18, 19]. Nevertheless, the optimized strategy for suprarenal cross-clamping and temporary renal perfusion has to be still defined and investigated [20–22].

A low-profile extracorporeal circulation (LPECC) strategy with a pressure- and volume-controlled system for selective renal perfusion during suprarenal cross-clamping was evaluated and monitored during open surgery. The main focus was the assessment of renal function during Intensive Care Unit (ICU) stay, initial the 30-day interval and during 1-year follow-up.

## Methods

### Patient population

Between October 2018 and January 2024, a total of 23 patients underwent surgery for JAAA at our institution. The inclusion criteria comprise diameter of JAAA ≥ 55 mm and a neck < 10 mm and/or complex necks involving the renal arteries, which were unsuitable for standard EVAR according to the current guidelines of the ESVS [14]. Terminal renal failure, significant stenoses of the renal arteries and previous abdominal surgery or EVAR were defined as exclusion criteria. Strategy for open surgery aimed for suprarenal clamping with juxtarenal anastomosis and avoidance of bypasses to the renal arteries. The patients were connected to the LPECC intraoperatively, with selective cannulation and perfusion of the renal arteries. All procedures were performed in accordance with the ethical standards of the institutional and/or national research committee and with the 1964 Helsinki declaration and its later amendments or comparable ethical standards. The study was submitted to and approved by the Ethic committee of the University Hospital of Schleswig-Holstein (D495/19). Informed consent to participate was obtained from all of the participants in the study.

### Study design

In this observational trial, following the STROBE checklist, we retrospectively evaluate in a consecutive case series the effect of pressure- and volume-controlled renal perfusion on clinical outcome and incidence of postoperative AKI after open surgery for JAAA [23].

The demographic, perioperative and postoperative variables were compared with the current literature. Postoperatively, an ultrasound-guided flow measurement of both renal arteries (ml/min) was performed in the ICU. The RIFLE classification (risk, injury, failure, loss, end-stage renal disease) was used as a standardized evaluation system to classify postoperative renal dysfunction [24]. Changes in the estimated glomerular filtration rate (eGFR) are categorized in the RIFLE criteria as follows: risk (eGFR decrease > 25%), injury (eGFR decrease > 50%), failure (eGFR decrease > 75%), loss (complete loss of kidney function > 4 weeks) and ESKD (end-stage renal disease with complete loss of kidney function for > 3 months). The definition of AKI was defined as a decrease in eGFR of more than 50% (RIFLE class ≥ 2) [25, 26]. Outcome parameters included postoperative retention parameters, the occurrence of AKI, the length of in-hospital stays and occurrences of complications. During follow-up, the most important parameters for AKI were temporary dialysis after 30-days and after 1-year [27, 28].

### Surgical procedures

**Fig. 1:**
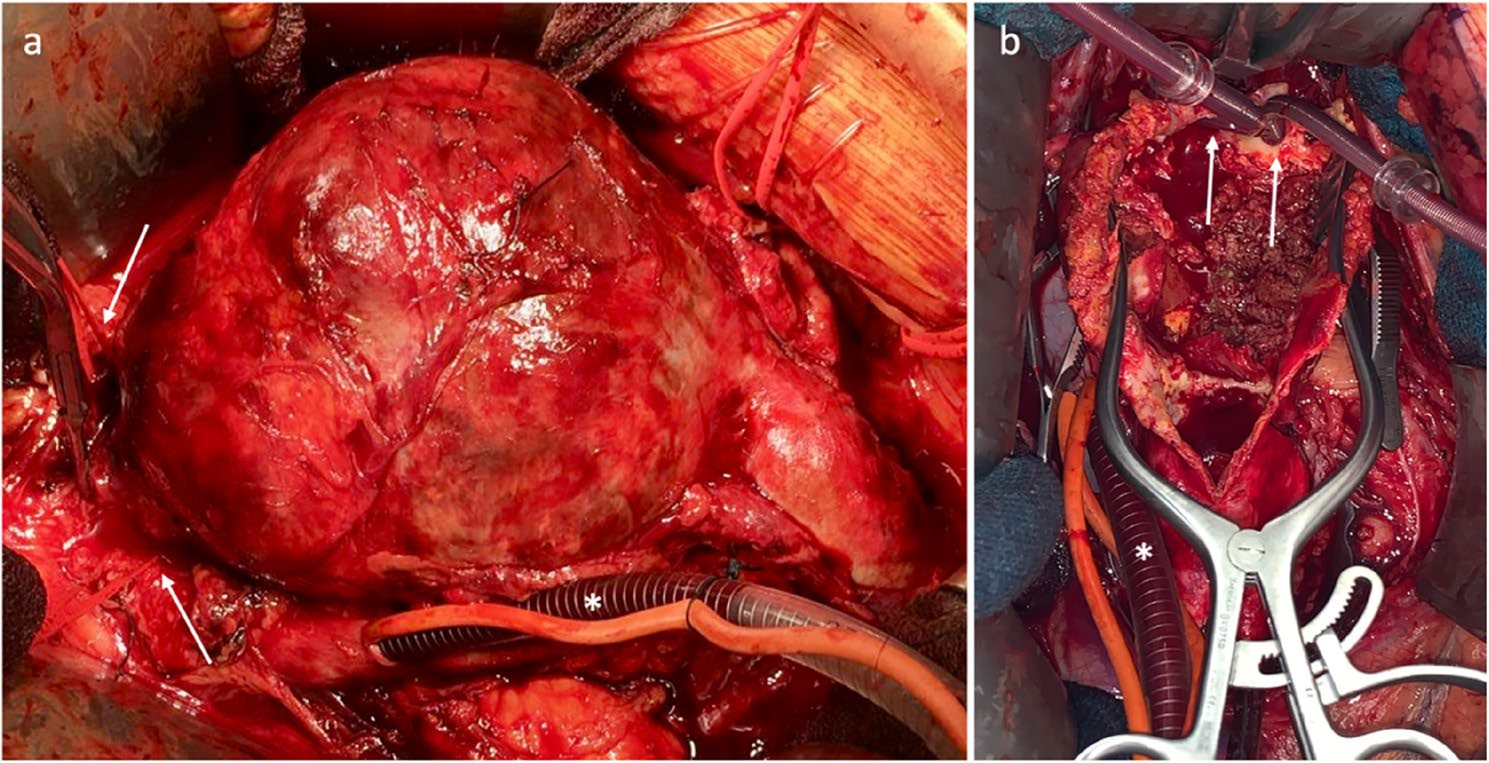
Intraoperative findings of a juxtarenal aortic aneurysm. **a** Selective cannulation of the ICV (asterisk) and both renal arteries (arrows) under suprarenal cross-clamping. **b** Opened aneurysm with selective perfusion of the right and left renal artery (arrows)

Surgery was performed under general anesthesia via median laparotomy and by a specialized “aortic cardiovascular team” at the University Hospital of Schleswig-Holstein, Campus Kiel. Replacement of the aorta was performed by implantation of a polyester graft e.g. aortic or bi-iliac prothesis. During suprarenal cross-clamping, renal perfusion was performed by selective cannulation via LPECC [Fig. 1]. Intraoperatively, all patients received systemic full heparinization controlled systematically by active clotting time (ACT). Heparin-induced ACT of 350–450 s was targeted during the cross-clamping period (300 IU/kg body weight). This increased ACT is necessary because of the risk of clotting of the oxygenator. Cannulation of the renal arteries was performed with flexible 8–10 French DLP™ pediatric arterial cannulas (Medtronic GmbH, Meerbusch, Germany). After completion of the proximal anastomosis, sequential distal prosthetic clamping and release of blood flow over the renal arteries was performed. If it was necessary to separate the left renal vein for better exposure, an interposition was performed after successful aortic replacement.

### Application of a low-profile extracorporeal circulation (LPECC) system

**Fig. 2:**
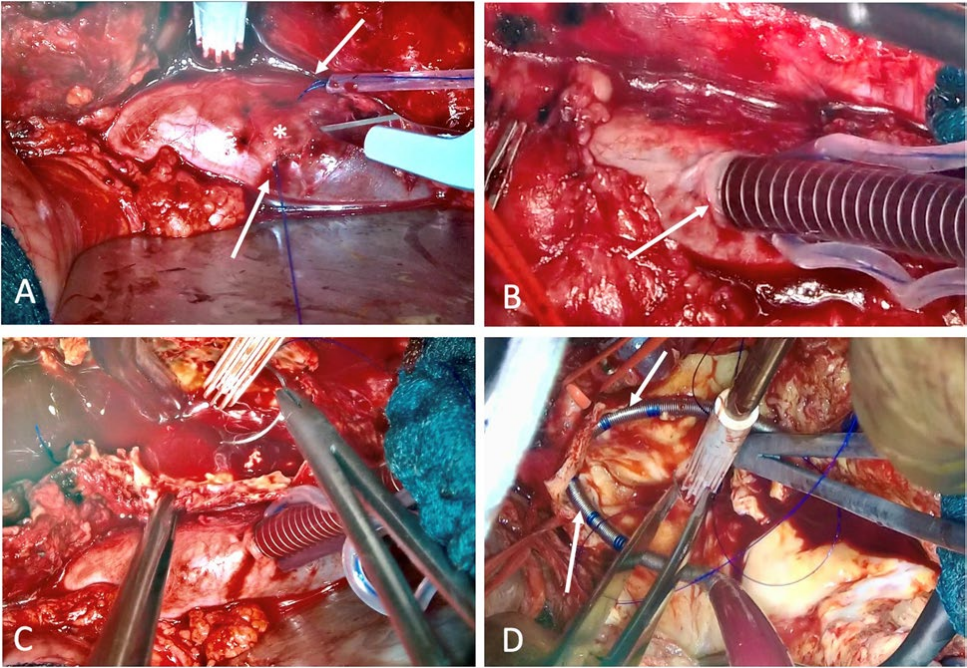
Illustration of the individual cannulation steps: (**a**) placement of two circumferential sutures (arrows) on the ICV (asterisk), (**b**) cannulation of the ICV (arrow), (**c**) opening of the aneurysm sac, (**d**) cannulation of the renal arteries (arrows)

After systemic heparinization LPECC was connected via cannulation of the inferior vena cava (IVC) using a 16–20 French cannula for venous return (V122 venous return cannula, LivaNova, London, United Kingdom) [Fig. 2]. During suprarenal cross-clamping, selective cannulation of the renal arteries with warm blood flows between 180 and 280 ml/min and mean arterial blood pressures ≥ 60 mmHg was performed using flexible 8–10 French DLP™ pediatric arterial cannulas and oxygenator (CAPIOX^ฏ^ FX05, Terumo Germany GmbH, Eschborn, Germany). During LPECC support pressure- and volume-controlled flow measurement (ELSA^ฏ^-Monitor, Transonic systems Inc., New York, United States) was continuously monitored by perfusionist directly at the outflow of the perfusion tubes. In addition, near-infrared spectroscopy (NIRS) registered regional changes of kidney perfusion in the lumbar region during cross-clamping. Intraoperative blood losses were directly retransfused using the LPECC via the renal cannulas.

### Statistical analysis

All statistical analysis was performed with the PRISM 10.2.3 software (GraphPad, San Diego, United States). Due to the nature of the present study, the results have been presented as descriptive data. Values of continuous data are presented as mean ± standard deviation. Categorical variables are presented as frequency distributions (n) and simple percentages (%).

## Results

**Fig. 3:**
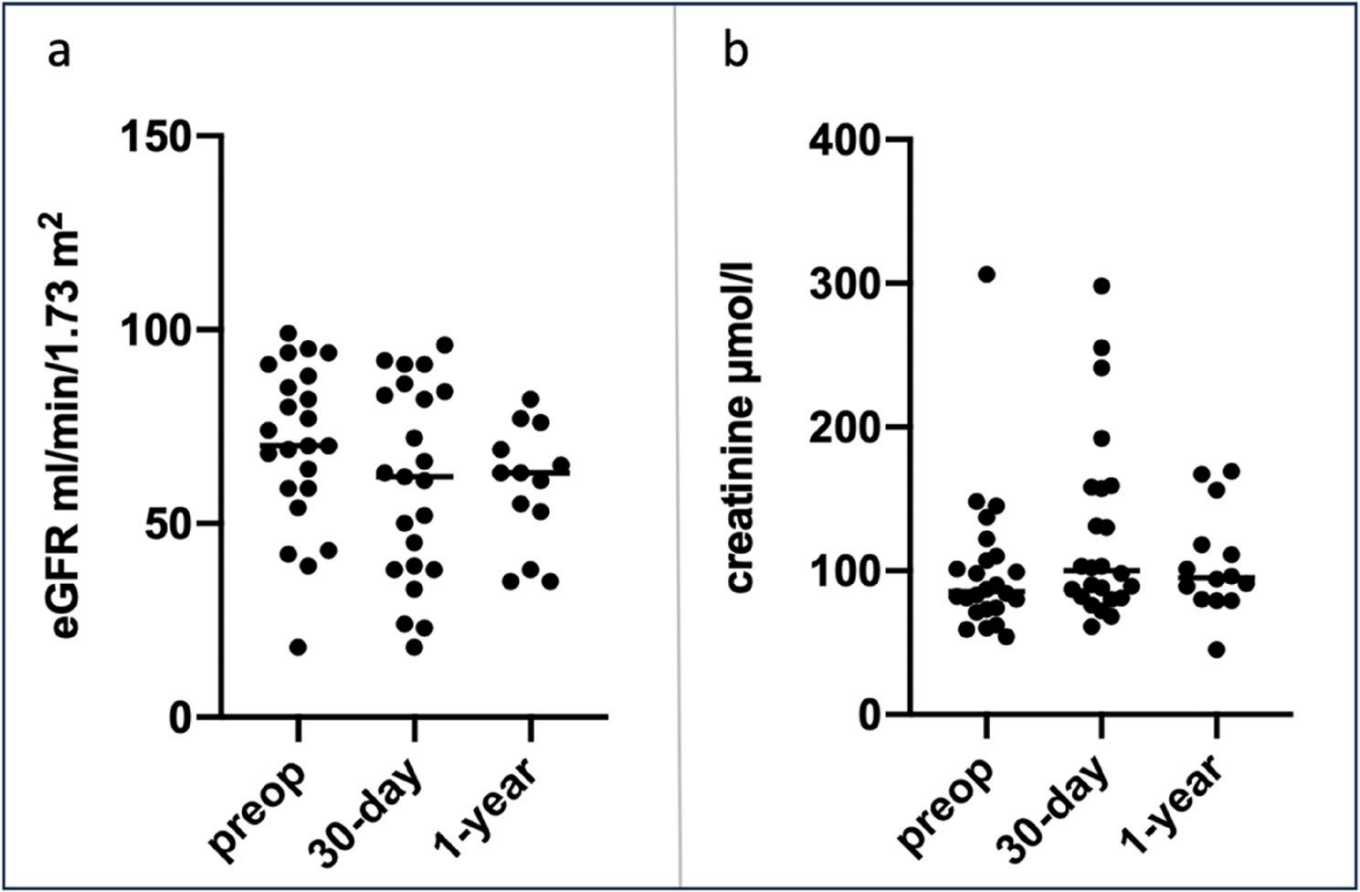
Evaluation of eGFR (**a**) and creatinine (**b**) during the study period

Intraoperative cannulation of both the IVC and renal arteries was feasible and without complications in all patients. There were 18 male patients (78%) and 5 female patients (22%) with a mean age of 68.43 ± 8.17 years [Table 1]. The most frequent comorbidities were arterial hypertension (70%), smoking (70%) and hyperlipoproteinemia (52%). None of the patients had terminal renal failure prior to surgery. One patient had a higher degree of renal impairment with significantly reduced eGFR (Fig. 3) During cross-clamping, average blood flow over LPECC was 216.74 ± 26.11 ml/min. Cross-clamping time was 29.52 ± 16.1 min with an unprotected ischemic time of 5.61 ± 3.38 min and renal perfusion time of 24.26 ± 15.51 min [Table 2]. Out of the 23 patients a bifurcated graft was used in 18 cases and a tube graft in 5 cases. None of the patients required a bypass to the renal artery.

**Table 1:**
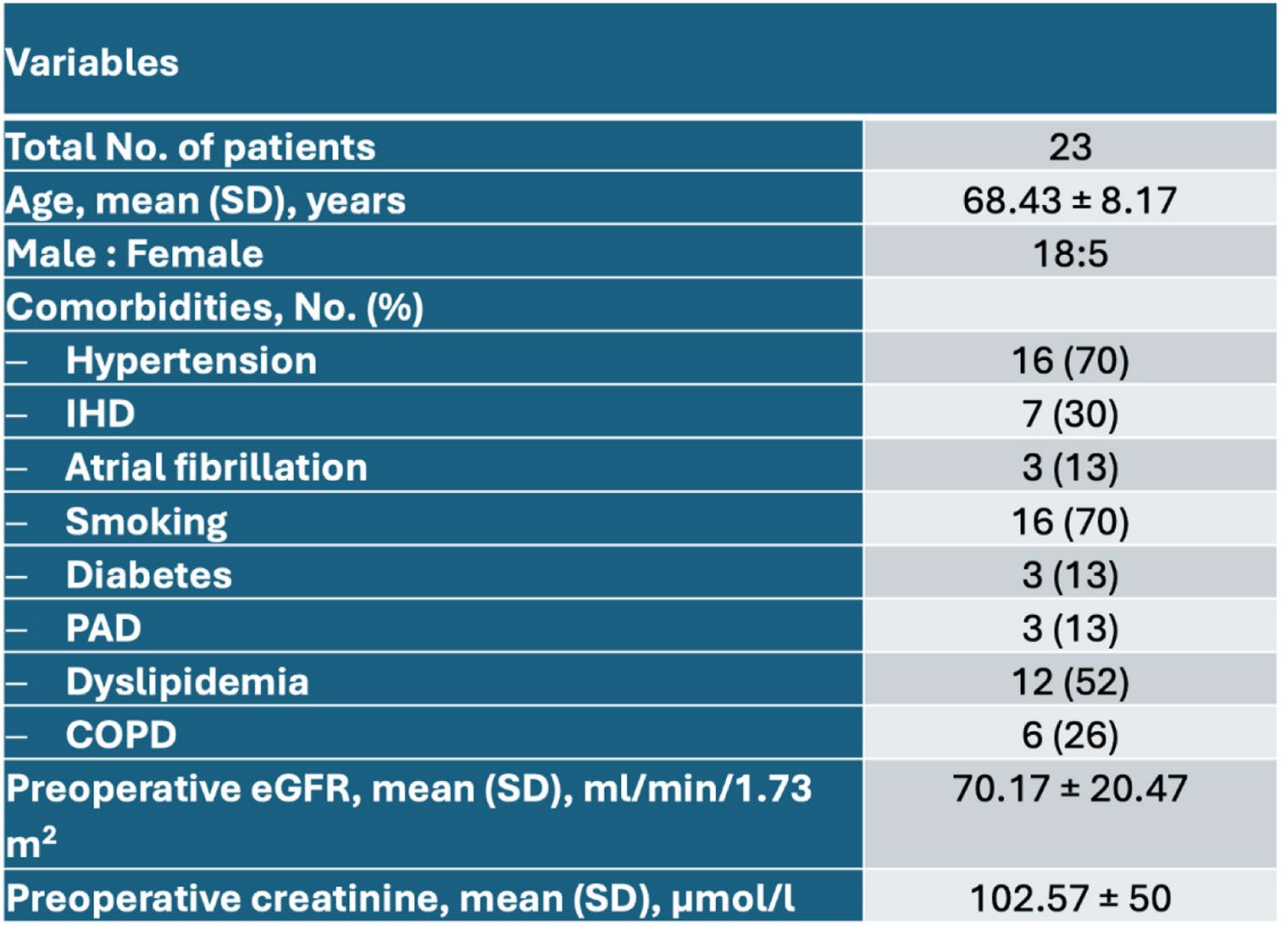
Baseline characteristics and comorbidities

*IHD* ischemic heart disease, *CKD* chronic kidney disease, *PAD* peripheral artery disease, *COPD* chronic obstructive pulmonary disease, *eGFR* estimated glomerular filtration rate

**Table 2:**
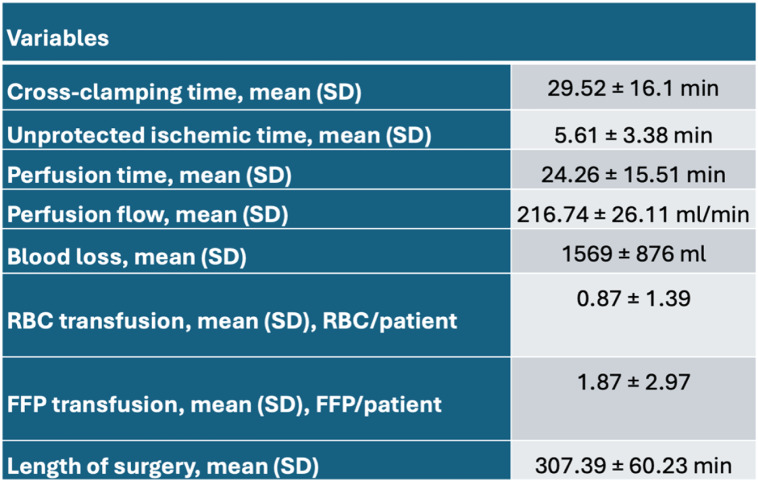
Operative details

*RBC* red blood cell, *FFP* fresh frozen plasma

The mean operative time was 307.39 ± 60.23 min with a mean intraoperative blood loss of 1569 ± 876 ml and a mean red blood cell (RBC) transfusion unit (200 ml) of 0.87 ± 1.39 RBC/patient. In the postoperative period, the ventilation time on ICU was 6.5 ± 5.72 h with a mean ICU-stay of 2.65 ± 2.1 d and a total in-hospital stay of 11.87 ± 6.15 d [Table 3]. The occurrence of renal dysfunction was 48% overall (*n* = 11), of which 64% showed mild dysfunction (RIFLE class 1, *n* = 7), 18% moderate dysfunction (RIFLE class 2, *n* = 2) and 18% severe (RIFLE class 3, *n* = 2) dysfunction. The majority of 91% were only temporary, with a return to normal retention values at discharge. Accordingly, 17% developed acute kidney injury (RIFLE class ≥ 2, *n* = 4) of which 3 patients required dialysis, which was temporary in the majority of cases (2/3). The analysis of the postoperative retention parameters showed an improvement in the 30-day follow-up for creatinine and eGFR, with recompensed laboratory values corresponding to the initial values. In the ICU, a standard sonography with flow measurement of both renal arteries was performed immediately postoperatively, which showed a regular perfusion flow in all patients. The documentation of the postoperative retention values showed a minimum of values eGFR of 41.0 ± 25.36 ml/min/1.73m^2^. In the further 30-day and 1-year follow-up mean values for the eGFR of 60.39 ± 24.36 ml/min/1.73m^2^ and 59.38 ± 15.08 ml/min/1.73m^2^ were observed and there was no significant difference to base values. The maximum increase in creatinine in the postoperative course was 225.65 ± 185.66 µmol/l. In the 30-day follow-up, the creatinine values were almost equal to the initial physiological values at 126.7 ± 63.42 µmol/l. In the 1-year follow-up, retention parameters were similar to the preoperative baseline values. Detailed analyses of the eGFR and creatinine after 30-days and 1-year did not identify any significant changes compared to the preoperative values [Fig. 3]. In the postoperative course, one patient died in multi-organ failure due to cardiac complications.

**Table 3:**
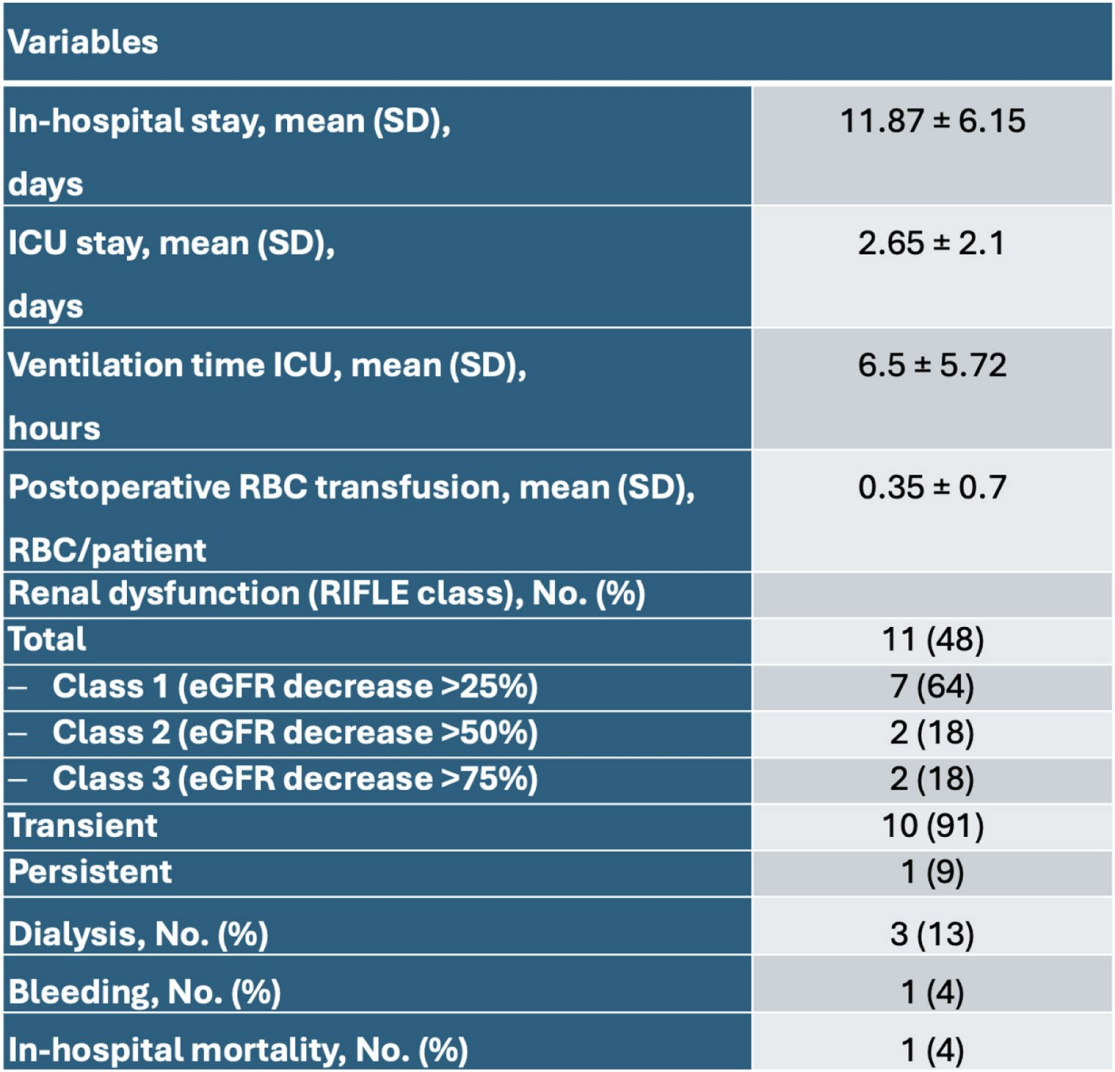
Postoperative results

*RBC* red blood cell, *ICU* intensive care unit, *RIFLE *(risk, injury, failure, loss, end-stage renal disease), *eGFR* estimated glomerular filtration rate

## Discussion

Nowadays, treatment of JAAA remains a major interdisciplinary challenge and the optimized therapeutic approach is often difficult to define [19]. A review of the current guidelines revealed that endovascular procedures for JAAA are a possible treatment option depending on anatomic, morphometric and clinical considerations [14, 29]. Nevertheless, there remains a relevant proportion of patients who are not feasible for endovascular treatment [25, 30]. In addition, several studies indicate that the increased use of endovascular procedures is accompanied by a correlating number of reinterventions [30, 31]. The current UK-COMPASS study demonstrated in a multicenter analysis of patients undergoing endovascular treatment of JAAA a worse midterm survival in presence of a short or complex neck [4].

Open surgery with aortic cross-clamping often results in organ damage, which can be increased by postoperative volume shift and complications [21, 32]. This raises the question which surgical techniques could preserve especially renal function in JAAA and whether this improves overall postoperative outcome.

The patients included in this study averaged an age of 68.43 ± 8.17 years, comparable to the literature references. Comorbidities were similar, with a typical set of comorbidities. The use of the LPECC was safely feasible without any intraoperative complications in all patients (*n* = 23). In a retrospective analysis, Pearce et al. (2007) demonstrated that in 678 patients with juxta- or suprarenal aneurysms undergoing open surgery, AKI developed in 14% of cases and required temporary dialysis in 7% of cases [33]. Other study groups reported that reduced preoperative renal function is strongly associated with increased postoperative mortality, with temporary or permanent postoperative renal replacement therapy further increasing mortality [10, 11]. In the here presented study, 17% (RIFLE class ≥ 2, *n* = 4) of the patients developed AKI, including three patients requiring dialysis which is consistent with the corresponding literature [22, 25, 26, 33]. During the clamping period, we were able to apply continuous flow over the renal arteries with controlled pressure intraoperatively using LPECC. Targeted renal perfusion allowed us to reduce the ischemic time and we assume that we were also able to increase ischemia tolerance as a result. Renal perfusion was measured by sonography as standard in the ICU and showed a regular renal perfusion in all patients. Accordingly, the AKI was not caused by perioperative reduced major renal perfusion. During 30-day follow-up, only 4% (*n* = 1) of patients still required dialysis, with the median retention parameters returning to the preoperative baseline values and after 1-year, no patient required dialysis with retention values comparable to the initial physiological values. It should be noted at this point that perioperative renal dysfunction is often multifactorial in origin and, in addition to intraoperative ischemia, is also significantly influenced by factors such as hemodynamic, volume status, and nephrotoxic medications. Furthermore, it is notoriously difficult to quantify the extent of perioperative renal dysfunction precisely or to assess validly whether the functional impairment is temporary or permanent, due to its multifactorial nature.

Due to the small patient cohort, this study did not specifically investigate the correlation between quantitative renal perfusion via extracorporeal circulation or the NIRS values recorded and the postoperative renal function observed.

Increasing experience in the application of surgical techniques as well as a dedicated aortic team including perfusionist and cardio-anaesthesiologists could certainly improve the operative times as well as the individual operative steps and thus reduce the perfusion times [14]. The ESVS guidelines support the centralization of complex aortic pathologies in specialized high-volume centers, describing that the experience of the center and the correlating level of surgical training play an essential role for postoperative outcome [14, 34]. Different research groups explicitly point out the connection between intraoperative ischemic time and the development of a postoperative AKI [32, 35]. Accordingly, the monitoring of intraoperative renal perfusion during LPECC plays a more important role in the assessment of ischemic time. Targeted monitoring of blood flow and indirect perfusion measurement using NIRS allows immediate adjustment of LPECC perfusion. Relative changes of NIRS indirectly indicate an insufficient renal perfusion, which could be compensated by an increased blood flow rate over the LPECC. During cross-clamping, the implantation and use of LPECC and renal perfusion after a short learning curve neither interfered with the creation of the proximal anastomosis nor prolonged the clamping and renal perfusion time. These results prove that the use of an LPECC is at least a comparable surgical method in the treatment of JAAA compared with previously established procedures. LPECC may provide enhanced organ protection through flow- and pressure-controlled perfusion. Another potential advantage is the immediate return of intraoperative blood loss via extracorporeal circulation which is superior to two-stage substitution using a cell saver especially in cases of short-term higher blood loss. Even though the small number of cases here does not allow for any definitive conclusions, this could result in a reduced demand for blood and coagulation products and thereby secondary reduction of the risk of AKI. However, the use of extracorporeal circulation carries specific risks, such as an increased bleeding risk due to anticoagulation even though we did not observe any significant intraoperative bleeding events in our cohort.

Moreover, cannulation of the visceral and renal vessels also requires the utmost care to avoid dissections and subsequent organ ischemia. There is also the possibility of access trauma when inserting the cannula into the inferior vena cava. On the other hand, cannulation of the IVC spares groin cannulation and associated complications. Overall, the use of extracorporeal circulation naturally entails additional logistical and financial costs and is associated with a longer operation time. On the other hand, we consider this effort to be justified in view of the potential benefit for the patient. Furthermore, the longer operation time does not affect the clamping phase itself, which is crucial for secondary organ damage.

Chiesa et al. (2006) reported that suprarenal cross-clamping time of ≥ 30 min can lead to a reduction in renal function [15, 18]. This study showed comparable cross-clamping times of 29.52 ± 16.1 min, without increased dialysis requirement in the study population, which is incoherent with the corresponding literature [15, 18]. In 2017 Sato et al. described the relevance of perfusion pressure for the development of AKI [36]. Renal perfusion pressure shows to be the most important intraoperative variable for possible kidney protection during cross-clamping [37, 38]. Compared to conventional renal protection techniques, LPECC enables continuous monitoring of renal perfusion and perioperative adjustment of volume flow and pressure ratios in response to potential organ undersupply. In the 30-day outcome of this study, a total of three patients were treated with temporary dialysis with two patients recovering their retention parameters to a level where they did not require further dialysis. These incidence of perioperative renal dysfunction is low in comparison to other JAAA case series [15, 18].

A major advantage of the LPECC is the open system, which allows continuous pressure and volume-controlled perfusion with simultaneous re-transfusion of intraoperative blood loss. The direct re-transfusion by the LPECC enables improved blood management with reduced need for blood components such as coagulation factors and reduced RBC and coagulation products in the postoperative course. Various studies have shown that controlled patient blood management (PBM) can reduce complication rates and thus to an improved outcome [39, 40]. The use of LPECC reduced blood products intraoperatively to 0.87 ± 1.39 RBCs/patient through direct re-transfusion of the blood loss. Effective transfusion strategies reduced the volume therapy and the use of vasopressor medication in the postoperative course and the patients could be transferred from the ICU to the peripheral ward after a median of 2.65 ± 2.1 days. Traina et al. (2020) confirm the importance of intraoperative volume management in patients with suprarenal cross-clamping as they usually show an increased volume and vasopressor usage compared to aortic procedures without suprarenal cross-clamping [41]. By reduced blood transfusions, avoidance of postoperative bleeding, and optimized volume therapy, a slight benefit in terms of mortality rate and complication rate seems to appear by using LPECC. Moreover, the current study showed a slight reduction of the ICU stay and the in-hospital stay using a LPECC [1, 14].

El-Sabrout et al. (2001) described suprarenal cross-clamping as a safe method for the controlled treatment of JAA [19]. These statements could be supported by this study and show the possibility to apply well-known strategies in new approaches under improved methods. In particular, the possibility of monitored pressure- and volume-controlled renal perfusion provides a safe method for the management of complex aortic pathologies. However, in view of the high level of equipment required and the necessary specialization of the interdisciplinary team, the use of LPECC should be restricted to high-volume aortic centers.

The main limitations of this study are the small patient cohort and the lack of a control group, both for conventional surgical techniques and for endovascular treatment using fenestrated prostheses. The patient selection is also based on the “fit for surgery” principle. Furthermore, the results regarding kidney protection and the general 30-day outcome are comparable to conventional surgical techniques and endovascular procedures. In this respect, the hypothesis postulated here of potentially better organ protection using the LPECC technique needs to be confirmed by following prospective studies with larger patient groups and control groups.

## Conclusions

Controlled blood loss via active re-transfusion by using LPECC may reduce both the need for red blood cell transfusion and the increased use of coagulation substitution in the postoperative period. The use of LPECC in the treatment of complex aortic aneurysms with extended suprarenal clamping has the potential to improve outcomes, especially in terms of renal function. Further prospective randomized studies for complex aortic repair with LPECC are mandatory to confirm the hypotheses put forward here regarding potential benefits such as nephroprotection and blood volume management. Until then, observations on the use of the extracorporeal circulation in complex aortic surgery can be regarded as proof of concept with potential benefits.

## Data Availability

The complete datasets used and analysed during the current study are available from the corresponding author on reasonable request.
